# Underreporting of adverse events to health authorities by healthcare professionals: a red flag-raising descriptive study

**DOI:** 10.1093/intqhc/mzae109

**Published:** 2024-11-28

**Authors:** Maude Lavallée, Sonia Corbin, Pallavi Pradhan, Laura Blonde Guefack, Magalie Thibault, Julie Méthot, Anick Bérard, Marie-Eve Piché, Fernanda Raphael Escobar Gimenes, Rosalie Darveau, Isabelle Cloutier, Jacinthe Leclerc

**Affiliations:** Faculty of Pharmacy, Université Laval, Quebec, Quebec G1V 0A6, Canada; Centre de Recherche de l’Institut Universitaire de Cardiologie et de Pneumologie de Québec- Université Laval, Quebec, Quebec G1V 4G5, Canada; Faculty of Pharmacy, Université Laval, Quebec, Quebec G1V 0A6, Canada; Centre de Recherche de l’Institut Universitaire de Cardiologie et de Pneumologie de Québec- Université Laval, Quebec, Quebec G1V 4G5, Canada; Centre de Recherche de l’Institut Universitaire de Cardiologie et de Pneumologie de Québec- Université Laval, Quebec, Quebec G1V 4G5, Canada; Department of Anatomy, Université du Québec Trois-Rivières, Trois-Rivières, Quebec G8Z 4M3, Canada; Centre de Recherche de l’Institut Universitaire de Cardiologie et de Pneumologie de Québec- Université Laval, Quebec, Quebec G1V 4G5, Canada; Department of Anatomy, Université du Québec Trois-Rivières, Trois-Rivières, Quebec G8Z 4M3, Canada; Centre de Recherche de l’Institut Universitaire de Cardiologie et de Pneumologie de Québec- Université Laval, Quebec, Quebec G1V 4G5, Canada; Faculty of Pharmacy, Université Laval, Quebec, Quebec G1V 0A6, Canada; Centre de Recherche de l’Institut Universitaire de Cardiologie et de Pneumologie de Québec- Université Laval, Quebec, Quebec G1V 4G5, Canada; CHU Sainte-Justine Research Center, Montreal, Quebec H3T 1C5, Canada; Faculty of Pharmacy, Université de Montréal, Montreal, Quebec H3C 3J7, Canada; Centre de Recherche de l’Institut Universitaire de Cardiologie et de Pneumologie de Québec- Université Laval, Quebec, Quebec G1V 4G5, Canada; Faculty of Medicine, Université Laval, Quebec Quebec G1V 0A6, Canada; Faculty of Nursing, University of São Paulo, São Paulo, São Paulo 05508-220, Brazil; Institut Universitaire de Cardiologie et de Pneumologie de Québec, Quebec, Quebec G1V 4G5, Canada; Faculty of Pharmacy, Université Laval, Quebec, Quebec G1V 0A6, Canada; Institut Universitaire de Cardiologie et de Pneumologie de Québec, Quebec, Quebec G1V 4G5, Canada; Faculty of Pharmacy, Université Laval, Quebec, Quebec G1V 0A6, Canada; Centre de Recherche de l’Institut Universitaire de Cardiologie et de Pneumologie de Québec- Université Laval, Quebec, Quebec G1V 4G5, Canada

**Keywords:** adverse events, pharmacovigilance, drug safety, pharmacoepidemiology, Vanessa law, underreporting

## Abstract

**Background:**

An adverse event (AE) is any undesirable medical manifestation in an individual who has received pharmacological treatment. To be considered serious (SAE), it needs to meet minimally one of the severity criteria by Health Canada. The most recent data (2006) suggested that AEs were underreported (<6%) to health authorities. In Canada, since the implementation of Vanessa’s Law (2019), hospitals are required to report SAEs; however, this law remains relatively unknown. The objectives of the study were: (i) to document the incidence of any AE and SAE over time in a ‘real’ clinical context, (ii) to quantify SAEs reported to Health Canada, and (iii) to assess whether Vanessa’s Law has led to an increase in mandatory reporting to Health Canada.

**Methods:**

We carried out a descriptive retrospective study at the Institut Universitaire de Cardiologie et de Pneumologie de Québec-Université Laval, including 500 patients hospitalized between 1 January 2018 and 31 December 2021 and randomized into 4 cohorts (125 patients/year). Descriptive and comparative analyses were performed.

**Results:**

The characteristics of the cohorts were: 43.6% women; median age: 69 years (min–max: 21–96 years), number of comorbidities: 4 (1–12). During their hospitalization, patients consumed 18 different drug products (2–56) and the median of observed SAEs/patients was 0 (0–10) (total: 302). The overall percentage of SAEs reported to Health Canada was 0%, before and following the implementation of Vanessa’s Law.

**Conclusion:**

According to 500 electronic medical records, SAEs were underreported to Health Canada, even after the implementation of Vanessa’s law.

## Introduction

The safety of the population consuming drugs is currently an important issue worldwide [1], as demonstrated by most developed countries having their own approval system for drugs [2–6]. Most of these countries require market authorization holders to report any adverse event (AE) from clinical trials [5, 7, 8]. However, clinical trials are typically targeted at specific populations [5, 7] which explains why post-marketing drug safety in a ‘real’ clinical context (referring to the environment and conditions in which healthcare is provided) is essential. For many years, different countries made it mandatory to report different types of AEs; France [9], Australia [10], and the USA [11] have regulations governing the reporting of some AEs. However, to our knowledge, no country has enforced mandatory reporting.

Furthermore, even if there are different laws to regulate AE reporting, many countries face the problematic issue of underreported AEs: worldwide <6% of AEs are properly reported, excluding Quebec data [12]. This substantial reporting gap impairs health authorities’ ability to clearly assess the risk–benefit ratio of marketed drugs, making the analysis crucial, both before and after marketing to ensure patient safety.

Vanessa Young’s death [13] in 2000, exposed significant gaps in drug safety policies in Canada. On 16 December 2019, Bill C-17 [named ‘Protecting Canadians from Unsafe Drugs Act (Vanessa’s Law) Amendments to the Food and Drugs Act’] was passed [14]. This new law made it mandatory to report all serious adverse events (SAEs) that occur in a hospital setting to help Health Canada to better monitor the safety of marketed drugs [14]. Health Canada is an entity responsible for helping Canadians maintain and improve their health. It ensures that high-quality health services are accessible and work to reduce health risks [15]. In Canada, any undesirable medical manifestation arising in an individual who has received pharmacological treatment is considered an AE unless otherwise proven [14]. It is considered as SAE, if it meets at least one of the following criteria: (i) causing or prolonging hospitalization, (ii) causing a congenital abnormality, (iii) causing persistent or significant disability or incapacity, (iv) endangering the life of the patient, or (v) causing death [14]. To date, whether the implementation of Vanessa’s Law has led to improved SAE reporting remains unknown.

The objectives of this study were: (i) to document the incidence of AEs and SAEs over time in a ‘real’ clinical context in Quebec, Canada, (ii) to quantify SAEs reported to Health Canada among those that have occurred, and (iii) to assess whether Vanessa’s Law has led to an increase in mandatory reporting to Health Canada.

## Method

### Study design

This is a descriptive retrospective study.

### Setting

The study was conducted from 1 January 2018 to 31 December 2021 at the Institut Universitaire de Cardiologie et de Pneumologie de Québec-Université Laval (IUCPQ-ULaval). IUCPQ-ULaval a 338-bed capacity tertiary care teaching hospital specialized in cardiovascular, pulmonary, obesity, and metabolic diseases [16]. Data were collected between 2021/08 and 2023/09. The Ethics Board Certificate was obtained. Data were denominated and protected by the Privacy Act. The Director of Professional Services provided approval.

### Participants

Every adult patient hospitalized at the IUCPQ-ULaval during the targeted period was eligible for the study. Patients were excluded if not taking any medication or were participating in a randomized double-blind clinical trial. The specified 500 records were randomly selected by an archivist.

### Variables

All the demographic data, including age, sex, weight, height, body mass index (kg/m^2^), and comorbidities were considered. As this is a retrospective study, the focus was primarily on sex due to the unavailability of gender information. Sex refers to a biological attribute that is associated with physical and physiological features (male and female) [17]. Gender is defined by the Institute of Gender and Health, Canadian Institutes of Health Research (2012) as ‘the socially constructed roles, behaviours and identities of female, male and gender-diverse people.’ Comorbidities were listed by the combined scores of the Elixhauser and Charlson comorbidity indeces to predict 30-day mortality across ICD-9 and ICD-10 [18]. The date of admission and discharge, coronavirus disease (COVID-19) status, main diagnosis, and length of stay were collected. All the diagnoses were coded according to the International Classification of Disease (ICD-10-CA) [19] by the archivists. Information about drug products administered (prescribed or not) during hospitalization by a healthcare professional (HCP): product name, period of consumption (if related to an AE), and drug products related to an AE were collected. Regarding the AE; the start and end date, if it was reported to Health Canada, patient outcomes, and the severity according to the severity criteria of Health Canada were collected. To standardize AEs, the international Medical Dictionary for Regulatory Activities (MedDRA) terminology was used with two levels of hierarchical structure: (i) classifications by medical discipline (SOC-System Organ Classes) and, (ii) groups of high-level terms (HLGT) [20]. The first outcome was the incidence of an AE and SAE over time in a ‘real’ clinical context. The second outcome was the proportion of SAE reporting each year between 2018 and 2021 while the third one was to observe an increase in SAE reporting rates (2018–2021). An objective proof (such as a form or note from a HCP) confirming the SAE was needed to consider an SAE declared.

### Source of data and data extraction process

The episode of care was investigated using the full electronic medical record (EMR), hosted on the CristalNet^TM^ Platform (https://www.dcicristalnet.com/). Data has been extracted and gathered in a Research Electronic Data Capture (REDCap^TM^) by four rigorously trained members (S.C., M.L., P.P., and M.T.). Two of them are healthcare professionals (both were nurses, one with >12 years of experience, the other with 6 years of experience), while the other two have experience in pharmacovigilance. All four members of the extraction teams used Health Canada’s definition of an identified AE and SAE in EMR. Calibration tests were performed at the beginning and follow-ups were carried out during data extraction to maximize reliability of data extraction between all four members. After the extraction of all the data, three members (S.C., M.T., and M.L.) validated the totality of the information collected to minimize the risk of information bias (double check on key data entry and outliers, removing duplicates, and completion of missing data). The research database was structured in four different tabs: (i) patient, (ii) hospitalization, (iii) medications, and (iv) adverse events [21].

### Study size

Calculation of the sample size was based to meet our first objective [22]. Considering the number of patients hospitalized in our center annually (16 000/year) and an estimated rate of SAEs of 20% [1], of which only 5% would be reported to Health Canada [12], a sample size of 243 patients/year in order to obtain a rate of serious AE reported to Health Canada was needed corresponding to a 95% accurate (5% two-sided). Power calculated after extracting 500 was sufficient, so extraction was stopped.

### Statistical methods

Missing data were checked for the following variables: age, sex, body mass index (BMI), number of comorbidities, length of hospitalization, status of COVID-19, number of units visited, number of drug products consumed, number of AEs, AEs reported to Health Canada, and AE outcome. To assess whether data were normally distributed, Shapiro–Wilk and Kolmogorov–Smirnov tests were performed, and histograms were produced. The annual proportion of AEs and SAEs reported to Health Canada were calculated per cohorts and globally. Rates were estimated to determine the incidence of AEs and SAEs through the study period. Trends were analyzed using linear regression models. Nonparametric tests were used to assess the correlation between different elements and the number of AEs per patient. Correlations between the number of comorbidities and the number of AEs/patient were studied using Spearman’s rho and a Kruskall–Wallis test was used to compare the demographic characteristics of eight variables between the cohorts. The results were stratified according to the severity of the AE [4]. All analyses were performed with IBM SPSS Statistics for Windows (Version 29.0. Armonk, NY, USA: IBM Corp). A statistical significance threshold of 0.05 and 95% confidence intervals were used.

## Results

### Descriptive data

Missing data are presented in Supplementary Table S1. Demographic characteristics are described globally and by annual cohorts (Table 1). The most frequent diagnosis classified by ICD-10-CA in the 500 EMR was ‘acute myocardial infection - I21’ (*n* = 68, 13.6%) (Supplementary Fig. S1). More details about ICD-10-CA are presented in Supplementary Materials (Supplementary Figs S2–S5). There were 57 variables extracted without missing data for most important variables (Supplementary Table S1). In the end, a variable difference [statistically significant only for length of stay (*P* <.001) and number of units visited (*P* = .010)] between the four different cohorts was observed when it comes to the results concerning AE and SAE (Table 1). For six demographic characteristics (age, sex, BMI, comorbidities, medication, and AE/patient), the results of comparative tests between cohorts were considered statistically significant (Table 1). Globally, the most frequent MedDRA SOC by AE was ‘Cardiac disorders’ (10 007 541) (Supplementary Fig. S6), but they are also presented by year (Supplementary Figs S7–S10) as well as MedDRA HLGT (Supplementary Figs S11–S14) for more details.

**Table 1.** Patients’ demographic, hospitalization, and drug product characteristics by cohorts and globally.

	*P*-value^b^	All cohorts (*n* = 500)	Cohort of 2018 (*n* = 125)	Cohort of 2019 (*n* = 125)	Cohort of 2020 (*n* = 125)	Cohort of 2021 (*n* = 125)
**Demographics’ characteristics**						
Age (year), median (min–max; IQR)^c^	0.096	69 (21–96; 16.75)	72 (21–93; 18.00)	67 (25–93; 14.50)	70 (25–96; 17.00)	68 (21–93; 17.00)
Female, *n*) (%)^a^	0.409	218 (43.6)	62 (49.6)	49 (39.2)	54 (43.2)	53 (42.4)
Body mass index (kg/m^2^), median (min–max; IQR)^c^	0.636	28.07 (15.21–64.08; 8.69)	27.36 (17.31–48.22; 9.15)	28.23 (17.57–64.08; 9.10)	28.16 (15.21–54.70; 8.80)	27.96 (17.44–61.06; 7.58)
Comorbidities	0.225	4 (0–12; 3.0)	4 (0–11; 3.0)	4 (0–9; 3.0)	4 (0–12; 3.5)	4 (0–10; 4.0)
**Hospitalization**						
Length of stay (days), median (min–max; IQR)^c^	<0.001	3 (1–19; 3)	3 (1–19; 3)	3 (1–12; 3)	4 (1–14; 4)	3 (1–9; 3)
Units visited, median (min–max; IQR)^c^	0.010	2 (1–7; 2)	2 (1–7; 2)	2 (1–7; 2)	2 (1–6; 1)	2 (1–5; 1)
COVID-19 diagnosis, n (only 2020–21)	N/A	4	N/A	N/A	2	2
**Drug product**						
Drug product, total, *n*	N/A	9568	2334	2473	2477	2284
Drug product by patient, median (min–max, IQR)^c^	0.570	18 (2–56; 12)	18 (7–53; 11)	18 (2–51; 11)	18 (5–52; 10)	18 (5–56; 12)

a Sum of percentages may vary from 100% due to rounding.

b Kruskall–Wallis tests used for *P*-value.

c IQR: interquartile range.

#### Rates of SAE per person-years

**Figure 1:**
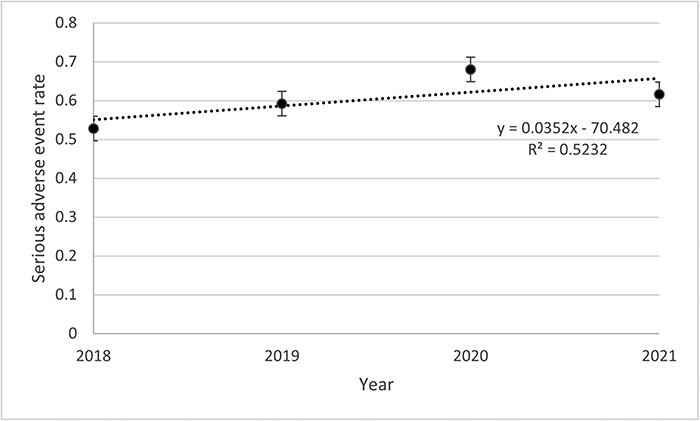
Annual SAE rates occurred at IUCPQ-ULaval.

**Figure 2:**
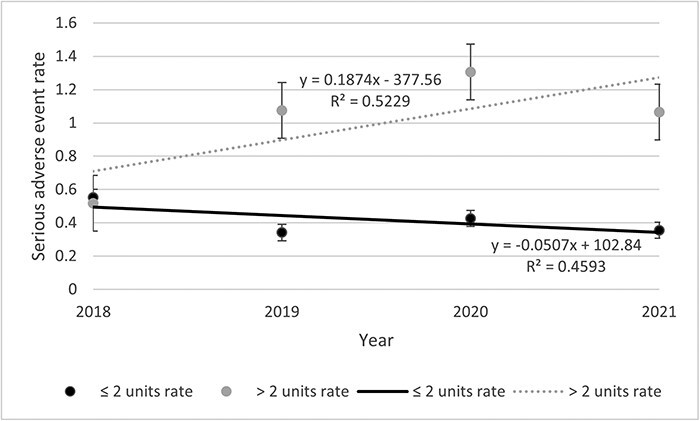
Annual SAE rates occurred at IUCPQ-ULaval, by years old.

**Figure 3:**
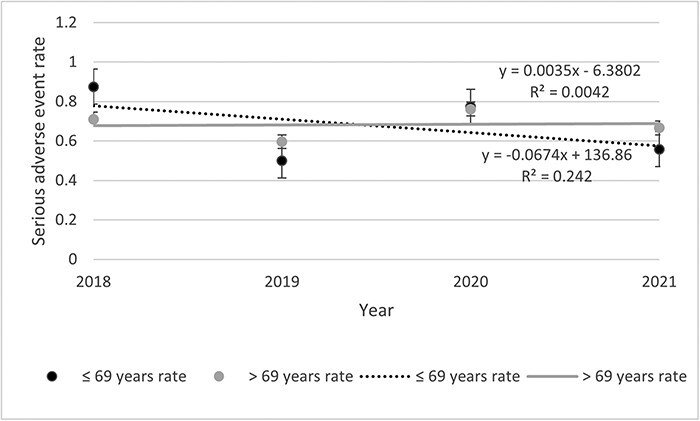
Annual SAE rates occurred at IUCPQ-ULaval, by units visited.

Table 2 presents data about the incident of any AE, SAE, and AE description. Among the 500 files, 2541 AEs including 302 SAEs were listed. The rates of SAEs remained stable throughout the study period (Fig. 1). The highest was observed in 2020 (0.68 SAE) and the lowest in 2018 (0.53 SAE). Rates of SAE decreased for the <69 years old (0.07%) while increasing for the >69 years old (0.004%) throughout the study period (Fig. 2). Then, if <3 units were visited, the rate decreased by 0.05% and increased by 0.19% if >3 units were visited (Fig. 3). Other stratifications are presented in Supplementary Materials (Supplementary Figs S15 to S19).

**Table 2.** Adverse events characteristics.

	All cohorts (*n* = 500)	Cohort of 2018 (*n* = 125)	Cohort of 2019 (*n* = 125)	Cohort of 2020 (*n* = 125)	Cohort of 2021 (*n* = 125)
AE, total, *n* (%)^a^	2541 (100)	675 (26.6)	679 (26.7)	619 (24.4)	568 (22.4)
AE, median (min–max; IQR)^b^	4 (0–40; 5)	4 (0–40; 6)	4 (0–23; 6)	4 (0–22; 5)	3 (0–28; 5)
Patient with 1 or more AE, *n* (%)^a^	458 (91.6)	118 (94.4)	113 (90.4)	113 (90.4)	114 (91.2)
Patient with no AE, *n* (%)^a^	42 (8.4)	7 (5.6)	12 (9.6)	12 (9.6)	11 (8.8)
AE reported to Health Canada, *n* (%)^a^	0 (0)	0 (0)	0 (0)	0 (0)	0 (0)
**Patient outcome**					
Recovering, *n* (%)^a^	336 (13.2)	154 (22.8)	85 (12.5)	53 (8.6)	44 (7.7)
Recovered, *n* (%)^a^	1609 (63.3)	382 (56.6)	443 (65.2)	376 (60.7)	408 (71.8)
Not recovered, *n* (%)^a^	88 (3.5)	26 (3.9)	25 (3.7)	18 (2.9)	19 (3.3)
Unknown, *n* (%)^a^	508 (20.0)	113 (16.7)	126 (18.6)	172 (27.8)	97 (17.1)
SAE, total, *n* (%)^a^	302 (8.4)	68 (22.5)	72 (23.8)	85 (28.1)	77 (25.5)
SAE, median (min– max; IQR)^b^	0 (0–10; 1)	0 (0–6; 1)	0 (0–7; 1)	0 (0–6; 1)	0 (0–10; 1)
Patient with 1 or more SAE, *n* (%)^a^	149 (29.8)	33 (26.4)	31 (24.8)	42 (33.6)	43 (34.4)
Patient with no SAE, *n* (%)^a^	351 (70.2)	92 (73.6)	94 (75.2)	83 (66.4)	82 (65.6)
SAE reported to Health Canada, *n* (%)^a^	0 (0)	0 (0)	0 (0)	0 (0)	0 (0)
**Severity criteria**					
Prolongation of current hospitalization, *n* (%)^a^	103 (34.1)	26 (38.2)	16 (22.2)	35 (41.2)	26 (33.8)
Congenital malformation, *n* (%)^a^	0 (0)	0 (0)	0 (0)	0 (0)	0 (0)
Persistent or significant disability, *n* (%)^a^	4 (1.3)	2 (2.9)	0 (0)	1 (1.2)	1 (1.3)
Incapacity, *n* (%)^a^	30 (9.9)	5 (7.4)	4 (5.6)	13 (15.3)	8 (10.4)
Life-threatening, *n* (%)^a^	148 (49.0)	31 (45.6)	44 (61.1)	34 (40.0)	39 (50.6)
Death, n (%)^a^	17 (5.6)	4 (5.9)	8 (11.1)	2 (2.4)	3 (3.9)
**Patient outcome**					
Recovering, *n* (%)^a^	40 (13.2)	7 (10.3)	13 (18.1)	11 (12.9)	9 (11.7)
Recovered, *n* (%)^a^	151 (50)	42 (61.8)	31 (43.1)	36 (42.4)	42 (54.5)
Not recovered, *n* (%)^a^	69 (22.8)	17 (25)	19 (26.4)	14 (16.5)	19 (24.7)
Unknown, *n* (%)^a^	42 (13.9)	2 (2.9)	9 (12.5)	24 (28.2)	7 (9.1)

a Sum of percentages may vary from 100% due to rounding.

b IQR: Interquartile range.

#### Rates of SAE reporting before and after the implementation of the Vanessa Law

Of the 302 SAE identified, none were reported to health authorities. No objective data were found to confirm the declaration of one of them. On the other hand, we were informed that between 16 December 2019 and 31 December 2021, 76 SAE out of 32 000 hospitalizations have been declared to Health Canada at IUCPQ-ULaval. We did not find these in our sample as this proportion of SAE declared represents 0.002%.

#### Rates of AE per person-year

The rate of AEs varied from 4.54 in 2021 to 5.40 per person-years in 2018 (Supplementary Fig. S20). The rate of AEs regarding length of stay followed two pathways: increased by 0.57 (2.82–3.39%) for length of ≤3 days rates and decreased by 0.99 (8.29–7.30%) for >3 days (Supplementary Fig. S21). All stratifications are presented in supplementary materials (Supplementary Figs S22–S26)

#### Rates of AE reporting over time

Of the 2541 AEs identified, no objective documentation was found to prove that any declaration was completed. Thus, the reported rates of AEs before and after the implementation of Vanessa’s Law remained at 0/person-year throughout the study period (2018–21).

### Other analyses

There was a correlation observed between the number of comorbidities and the number of AEs/patient (*r* = 0.0879; *P* = .009) (Supplementary Table S2). As the number of comorbidities increases, the occurrence of AEs also increases. There was a correlation between the number of drug products used (*r* = 0.619; *P* < .001) (Supplementary Table S3) and the length of hospital stay (*r* = 0.588; *P* < 0.001) (Supplementary Table S4) with the number of AEs.

## Discussion

### Statement of principal findings

We found that none of the SAEs identified by our research team following Health Canada’s definition in the 500 patients’ record were reported to Health Canada between 2018 and 2021. Furthermore, the implementation of Vanessa’s law had minimal impact on the mandatory reporting of SAEs. In fact, 76 SAEs reported by HCPs from IUCPQ-ULaval were found for the period 16 December 2019 to 31 December 2021 in an internal registry a posteriori, but there were not included in the 302 identified by our research team in this study. However, these 76 SAE reports indicate that while some HCPs are aware of SAEs, not all of them are. Another important point to raise is the need to validate that all HCPs recognize their role and responsibilities about reporting SAEs. To our knowledge, this retrospective intrahospital study is the first of its kind in Canada and highlights a gap to fill regarding population safety.

### Strengths and limitations

The retrospective nature of our study, conducted at a single tertiary academic center, raises concerns about generalizability and the possibility of information bias. Having full access to comprehensive hospitalization records through the EMR could have mitigated these risks. Considering the healthcare system context, conducting a prospective study would have been challenging. Our team had access to the EMR, but only for current hospitalizations relevant to our study, not comprehensive historical data. A confusion bias persists due to the COVID-19 pandemic, possibly influencing SAE reporting. However, looking at both pre- and post-pandemic, no AE or SAE reports surfaced. Random selection by an independent archivist minimizes selection bias. This study has several strengths by its innovative nature and results. Indeed, to our knowledge, this is the first Canadian intra-hospital study documenting the underreporting of AEs/SAEs to a health authority and to document the impact of Vanessa’s Law. In addition, the risk of selection bias was minimized through a random selection carried out independently by an archivist.

### Interpretation within the context of the wider literature

The underreporting of AEs remains an important issue that must be investigated [1, 23–26]. One study reported that 137 AEs were notified over a period of 5 years in a hospital center accounting for 80 842 medical visits to the clinic in addition to 3365 hospitalizations [26]. Another study observed a reporting rate of 7.4 AEs per 100 patients [23]. The Milan’s San Carlo Borromeo Hospital is a 72-bed medical ward that participated in a drug surveillance program called ARIES (Adverse Reactions Identification Evaluation System) in order to potentially increased awareness of HCPs [23]. A systematic literature review [12] that examined 37 studies from 12 different countries (between 1969–2004), including one from Canada, focused on the incidence of a specific AE (drug-induced toxic epidermal necrolysis) and reported an underreporting rate of 94% [12].

The different definitions of an AE may also explain the discrepancies between studies [23]. For example, the World Health Organization (WHO) defines an AE as ‘an injury related to medical management, in contrast to complications of disease,’ [27] while Health Canada includes all undesirable medical manifestations [28]. Using a similar retrospective methodology, the study by Maistrello et al. (1995) reported a similar proportion (110 AEs for 120 cases; 91.7%) with a proportion of SAEs was similar to our findings.

The Japanese Adverse Drug Event Report (JADER) database showed a considerable impact of the new legislation for vaccine-related spontaneous reporting (5.3 times more reports for a year relating to vaccination) [25]. The average number of reports per year passed from 231 reports to 1227 reports per year after the regulation changes. This contrasts with our findings as the implementation of Vanessa’s Law seems to have no impact on the number of reports of AEs. However, this Japanese study was interested in spontaneous reporting rather than those with mandatory signal detection performance characters [25]. Since the implementation of Vanessa’s Law, Canadian health authorities have made the numbers of reports received publicly available [29]. There were 20 866 AEs reported, including more than 15 000 SAEs in <4 years. In the province of Quebec, there were nearly 5000 mandatory reports. Quebec currently has 34 health institutions [30] grouping together 293 public hospital centers [31], which would equate toan approximate average number of 42 annual SAE declarations per health institution. However, according to our study, for 4 years observed, almost twice as many SAE would have occurred annually for a sample of only 125 patients, suggesting that an underreporting problem remains.

### Implications for policy, practice, and research

Our results concerning declarations are probably generalizable to other centers in the province of Quebec, Canada. Given that academic healthcare centers are generally more aware of new legislation and clinical guidelines [32] we could hypothesize that other centers lack sufficient resources to apply Vanessa’s Law requirements which can lead to implementation delays and indirectly impact the declaration of SEAs by HCPs. However, even if it is now mandatory nationwide to report SAEs for healthcare centers, there is no agreed upon standard by which to do so, either at the province or country level. Each healthcare center applies and decides the exact procedure they will use.

## Conclusion

No Canadian study, to our knowledge, has studied the occurrence trends for AEs and SAEs in an academic hospital center in a real-life episode of care setting. SAEs remain underreported worldwide, and this study also demonstrated that Quebec is no exception. HCPs undoubtedly play an important role in improving population safety and should be made more aware of the importance of reporting to better safeguard their patients. Neither an AE nor an SAE had been reported to Health Canada in the 500 files studied, despite it being mandatory to do since December 2019. However, we found 76 declarations in pharmacy registry after investigation. Even though a new law was passed to make the mandatory reporting of SAE, our study showed that the SAE occurring in the hospitals remains underreported. Upcoming studies should focus on knowledge of HCPs about new legislation and knowledge transfer programs to continue to raise awareness about the purpose of reporting. We considered that being aware of SAEs is the first step to an improvement of clinical practice.

## Supplementary Material

mzae109_Supp

## Data Availability

Authors will make available the data and program codes used in the analysis to any researcher for purposes of reproducing the results or replicating the procedure as Supplementary Material.
