# Supplementary material for: Underreporting of adverse events to health authorities by healthcare professionals: a red flag-raising descriptive study
Source: Int J Qual Health Care. 2024 Nov 28;36(4):mzae109. doi: 10.1093/intqhc/mzae109 (PMC11879413; doi:10.1093/intqhc/mzae109)
Supplement: mzae109_Supp [file mzae109_supp.zip › mzae109_Supp/suppl_data/Supplementary materials_IJQHC_16 décembre.docx]

**Supplementary materials**

**Table S1. Missing data by variables**

**^a^AE : Adverse event**

|  | All cohorts  (n=500) | Cohort of 2018  (n=125) | Cohort of 2019 (n=125) | Cohort of 2020 (n=125) | Cohort of 2021 (n=125) |
| --- | --- | --- | --- | --- | --- |
| Age | 0 | 0 | 0 | 0 | 0 |
| Sex | 0 | 0 | 0 | 0 | 0 |
| Body mass index | 30 | 15 | 4 | 6 | 5 |
| Number of comorbidities | 0 | 0 | 0 | 0 | 0 |
| Lenght of stay | 0 | 0 | 0 | 0 | 0 |
| COVID-19 positive | 62 | 0 | 0 | 31 | 31 |
| Units visited | 0 | 0 | 0 | 0 | 0 |
| Number of drugs consumed | 0 | 0 | 0 | 0 | 0 |
| Number of AE | 0 | 0 | 0 | 0 | 0 |
| Declared to Health Canada | 0 | 0 | 0 | 0 | 0 |
| Patient outcome | 0 | 0 | 0 | 0 | 0 |

**Table S2. Correlation test between the number of comorbidities and the number of AE**

| **Correlation** | | | | |
| --- | --- | --- | --- | --- |
|  | | | **Number of comobidities** | **Number of AE** |
| **Rho de Spearman** | Number of comorbidities | Correlation coefficient | 1.000 | .117^**^ |
|  |  | Sig. (two-sided) | . | .009 |
|  |  | N | 500 | 500 |
|  | Number of AE | Correlation coefficient | .117^**^ | 1.000 |
|  |  | Sig. (two-sided) | .009 | . |
|  |  | N | 500 | 500 |
| **. The correlation is significant at the 0.01 level (two-sided). | | | | |

**Table S3. Correlation test between the number of medication and the number of AE**

| **Correlations** | | | | |
| --- | --- | --- | --- | --- |
|  | | | Number of AE | Number of medication |
| **Rho de Spearman** | Number of AE | Correlation coefficient | 1.000 | .578^**^ |
|  |  | Sig. (two-sided) | . | <.001 |
|  |  | N | 500 | 500 |
|  | Number of medication | Correlation coefficient | .578^**^ | 1.000 |
|  |  | Sig. (two-sided) | <.001 | . |
|  |  | N | 500 | 500 |
| **. The correlation is significant at the 0.01 level (two-sided). | | | | |

**Table S4. Correlation test between the length of stay and the number of AE**

| **Correlation** | | | | |
| --- | --- | --- | --- | --- |
|  | | | Number of AE | Length of stay |
| **Rho de Spearman** | Number of AE | Correlation coefficient | 1.000 | .629^**^ |
|  |  | Sig. (two-sided) | . | <.001 |
|  |  | N | 500 | 500 |
|  | Length of stay | Correlation coefficient | .629^**^ | 1.000 |
|  |  | Sig. (two-sided) | <.001 | . |
|  |  | N | 500 | 500 |
| **. The correlation is significant at the 0.01 level (two-sided). | | | | |

**Figure S1. 10 most frequent diagnostic classified with ICD in 2018-2021**

**Figure S2. 10 most frequent diagnostic classified with ICD in 2018**

**Figure S3. 10 most frequent diagnostic classified with ICD in 2019**

**Figure S4. 10 most frequent diagnostic classified with ICD in 2020**

**Figure S5. 10 most frequent diagnostic classified with ICD in 2021**

**Figure S6. 10 most frequent AE classified with MedDRA SOC between 2018-2021**

**Figure S7. 10 most frequent AE classified with MedDRA SOC in 2018**

**Figure S8. 10 most frequent AE classified with MedDRA SOC in 2019**

**Figure S9. 10 most frequent AE classified with MedDRA SOC in 2020**

**Figure S10. 10 most frequent AE classified with MedDRA SOC in 2021**

**Figure S11. 10 most frequent AE classified with MedDRA HLGT in 2018**

**Figure S12. 10 most frequent AE classified with MedDRA HLGT in 2019**

**Figure S13. 10 most frequent AE classified with MedDRA HLGT in 2020**

**Figure S14. 10 most frequent AE classified with MedDRA HLGT in 2021**

**Figure S15. Annual serious adverse event rate occurred at IUCPQ-ULaval, by sex**

**Figure S16. Annual serious adverse event rate occurred at IUCPQ-ULaval, by comorbidities**

**Figure S17. Annual serious adverse event rate occurred at IUCPQ-ULaval, by length of stay**

**
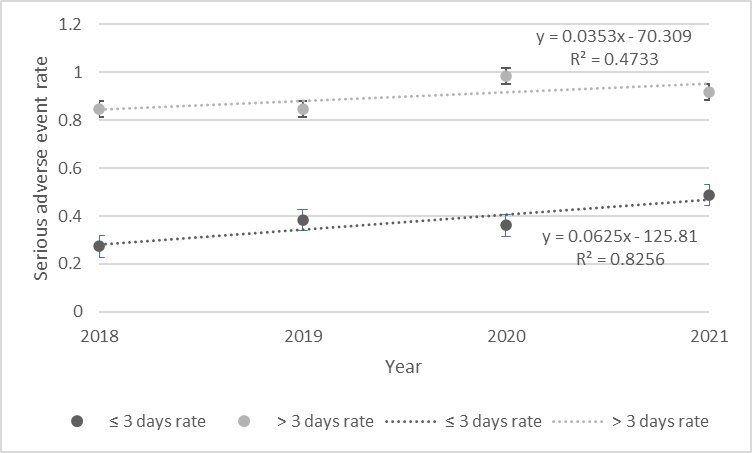
**

**Figure S18. Annual serious adverse event rate occurred at IUCPQ-ULaval, by number of drugs consumed**

**Figure S19. Annual serious adverse event rate occurred at IUCPQ-ULaval, by number of adverse event**

**Figure S20. Annual rate of incident AEs**

**Figure S21. Annual AE rate occurred at IUCPQ-ULaval, by length of stay**

**Figure S22. Annual rate of adverse events occured at IUCPQ-ULaval, by sex**

**Figure S23. Annual rate of adverse events occurred at IUCPQ-ULaval, by age**

**Figure S24. Annual adverse event rate occurred at IUCPQ-ULaval, by units visited**

**Figure S25. Annual adverse event rate occurred at IUCPQ-ULaval, by comorbidities**

**Figure S26. Annual adverse event rate occurred at IUCPQ-ULaval, by number of drugs consumed.**
